# SINR- and MI-Based Double-Robust Waveform Design

**DOI:** 10.3390/e24121841

**Published:** 2022-12-17

**Authors:** Fengming Xin, Jing Li, Yan Wang, Mingfeng Zhang

**Affiliations:** School of Computer and Communication Engineering, Northeastern University at Qinhuangdao, Qinhuangdao 066004, China

**Keywords:** signal-to-interference-plus-noise ratio (SINR), mutual information (MI), single-robust, double-robust

## Abstract

Owing to cognitive radar breaking the open-loop receiving–transmitting mode of traditional radar, adaptive waveform design for cognitive radar has become a central issue in radar system research. In this paper, the method of radar transmitted waveform design in the presence of clutter is studied. Since exact characterizations of the target and clutter spectra are uncommon in practice, a single-robust transmitted waveform design method is introduced to solve the problem of the imprecise target spectrum or the imprecise clutter spectrum. Furthermore, considering that radar cannot simultaneously obtain precise target and clutter spectra, a novel double-robust transmitted waveform design method is proposed. In this method, the signal-to-interference-plus-noise ratio and mutual information are used as the objective functions, and the optimization models for the double-robust waveform are established under the transmitted energy constraint. The Lagrange multiplier method was used to solve the optimal double-robust transmitted waveform. The simulation results show that the double-robust transmitted waveform can maximize SINR and MI in the worst case; the performance of SINR and MI will degrade if other transmitted waveforms are employed in the radar system.

## 1. Introduction

The concept of cognitive radar, first introduced in 2006 [1], is a symbolic representation of the new generation of radar systems. Cognitive radar [1,2] breaks the fixed mode of operation of traditional radar by introducing a closed-loop system. The radar transmitted waveform is adaptively transmitted through the analysis of the environment and the target information. It can enhance the performance of the radar system. Mutual information (MI) is an essential indicator for radar target estimation [3,4]. Bell was the first to employ the MI criterion for optimal waveform design [5], maximizing MI in a noise background to design the transmitted waveform. Then, a new information theory design method for a single emission waveform was proposed in [6], which extended Bell’s information-theoretic water-filling approach to allow the optimization of transmitted waveforms for multiple targets. In addition, the signal-to-interference-plus-noise ratio (SINR) is a significant metric of radar target detection performance [7,8,9]. Radar can improve its detection ability by maximizing the output SINR of the matched filter. In [10], the transmitted waveform design method for a known target and a stochastic extended target based on SINR and MI was investigated, and the relationship between SINR and MI was given in the context of waveform design for stochastic targets. The work in [11] combined the optimal waveform design based on SINR with the sequential hypothesis testing problem to form a closed-loop operation, which enabled the radar to change the next transmission waveform according to current environmental knowledge. As a result, the problems of radar adaptive waveform design and multi-target classification were solved. Li et al. studied the radar’s automatic identification systems to increase target recognition accuracy [12]. The particle swarm optimization (PSO) algorithm was investigated in [13,14,15]. Indeed, the PSO algorithm-based waveform design method can increase the estimation accuracy of radar systems [16]. The waveform design of radar and the extended target was developed in [17], and three different countermeasure models between smart radar and dumb target, smart target and dumb radar, and smart radar and smart target are proposed. Based on the minimum value theory, a new two-step water-filling approach was presented for the last situation. The work in [18] investigated the challenge of radar waveform design for recognizing doubly spread targets in colored noise under low signal power situations. By optimizing the Kullback–Leibler divergence, the detection performance of the detector is maximized. In [19,20], an optimal waveform and receiver design was introduced to solve the maximum SINR to improve target detection performance. The radar and communication systems coexist in a given band in the case of a joint radar communication system [21], and the optimal waveform matching the spectrum need was designed. In [22,23], the game model was considered to improve the capacity and security of communication networks. Bica et al. designed the radar waveform by identifying the communication signal scattered on the target as one of three cases: useful energy, interference, or completely ignored by the radar receiver [24]. The work in [25] proposed two hypotheses: the target is absent from the echoes and the target is present in the echoes. Maximizing the relative entropy between the two hypotheses enhances the detection performance and reduces the symbol error rates. In [26], the problem of cognitive radar waveform optimization design for multiple extended targets was explored. An improved algorithm was employed by maximizing the detection probability of the received echo, and the information theoretical approach was also taken into consideration under the same limitations on waveform energy and bandwidth.

However, the above research was based on a known target spectrum or clutter spectrum. Accurate target and clutter spectrums are difficult to obtain in complex electromagnetic environments due to a lack of prior knowledge of target and clutter. To address this problem, the joint optimization of the radar transmitted waveform and the receive filter was considered in [27,28]. An iterative optimization procedure was developed to realize the joint robust design of the transmitted waveform and receiving filter polarization. Similarly, to identify range-spread targets in the presence of clutter [29], iterative algorithms based on semi-definite programming (SDP) relaxation were devised to design radar waveforms combined with a filter array. Moreover, when considering the spectrum uncertainty, the robust radar transmitted waveform was designed in [30] based on [24], which took into account three instances of the communication signal scattered on the target. In [31], a radar waveform recognition algorithm based on random projections and sparse classification was presented to promote information completeness, efficiency, and noise robustness. For distributed multi-radar systems, the problem of robust waveform design based on the low probability of intercept (LPI) was addressed in [32], and a robust waveform design method based on LPI-SINR and LPI-MI criteria was proposed for the given system performance. In [33], an optimization model was established based on SINR and MI, in which the lower bound of the uncertain range of the target spectrum was taken as the target spectrum in the optimization problem to maximize the SINR and MI of radar and echo. A robust waveform design method based on harmonic variance and MI was proposed for detecting multiple targets in [34], and its performance was compared with that based on the original variance. In [35], the transmit beampattern design used an arbitrarily correlated linear frequency modulated (LFM) waveform set. For millimeter-wave radar [36], considering that the clutter spectrum is in an uncertain range, the optimization criterion is to maximize SINR and MI to guarantee the detection and recognition capabilities of the radar. Two kinds of robust transmitted waveforms were designed in the 135GHz–145GHz millimeter-wave band.

The design of a robust transmitted waveform only considers one of the cases of target spectrum fuzzy or clutter spectrum fuzzy. Our research team previously only considered single-robust radar waveform designs with an uncertain target spectrum [33]. In [33], the SINR- and MI-based maximin single-robust waveform design techniques were proposed, respectively. In this paper, we continue the in-depth study based on our previous research results in the literature [33]. The main contribution is that the uncertainty of the target and clutter spectra are both considered in designing the radar transmitted waveform. Firstly, the optimal transmitted waveform design methods based on SINR and MI are given under the condition of the certain target and clutter spectra. Secondly, for the two cases where the radar cannot accurately obtain the target spectrum or clutter spectrum, the single-robust radar waveform design methods based on the SINR and MI criteria are given. Then, for the case where both the target spectrum and the clutter spectrum are simultaneously in the corresponding uncertainty range, the double-robust radar waveform design methods based on the SINR and MI criteria are proposed. The Lagrange multiplier method is used to solve the optimization models for double-robust waveform design. The main idea is to introduce a new parameter (Lagrange multiplier λ) between the objective function and the constraint condition to solve the optimization models. 

The rest of this paper is organized as follows. In Section 2, the system model and criteria for waveform design are introduced. In Section 3, taking into account the uncertainty of spectrum estimation, the design scheme of a single-robust transmitted waveform is introduced. Then, for the situation where the uncertain target spectrum and the uncertain clutter spectrum exist simultaneously, the design of the double-robust transmitted waveform is proposed. In Section 4, the simulation results for three different robust models are shown, along with the associated analyses. Finally, in Section 5, the conclusions are presented.

## 2. System Model and Design Criteria

**Figure 1:**
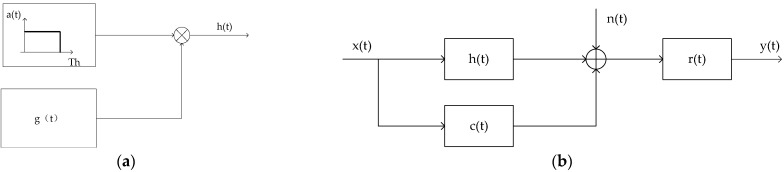
System and signal models. (**a**) Signal model for finite-duration random target. (**b**) Signal model for finite-duration random target in signal-dependent interference.

The model for the random target is given in Figure 1 [10]. Figure 1a shows the implementation process of a random target, where g(t) represents a generalized stationary random process and a(t) denotes a rectangular window function with duration Th. Therefore, the product h(t)=a(t)×g(t) represents a valued local stationary random process within [0, Th]. Figure 1b depicts the signal model for transmitted waveform design based on SINR and MI, where x(t) denotes the transmitted waveform signal and h(t) represents a finite-duration random process, and X(f) and H(f) denote the Fourier transforms of x(t) and h(t), respectively. r(t) represents the impulse response of the receiver filter, and n(t) is a zero-mean channel noise process with the power spectrum density (PSD) $S_{nn}\left(f\right)$. Likewise, c(t) represents an interference signal which is a zero-mean Gauss stochastic random process with the PSD $S_{cc}\left(f\right)$.

The energy spectrum variance (ESV) of a random target is written as Equation (1), which describes the average energy of the target signal in a finite duration.

(1) $$\sigma_{H}{}^{2}\left(f\right)=E\left[{\left|H\left(f\right)-\mu_{H}\left(f\right)\right|}^{2}\right]$$

where E{·} denotes the expectation of an input entity, and $\mu_{H}\left(f\right)$ represents the mean value of H(f) which is assumed to be 0.

To effectively improve the detection performance of the radar systems, the output SINR of the matched filter in the radar receiver is adopted as the criterion for designing the transmitted waveform. Maximization of SINR means the best radar detection performance. Thus, the expression for the SINR can be represented by [10]

(2) $$\;\mathrm{SIN}R\;=\int_{BW}\frac{\sigma_{H}{}^{2}\left(f\right){\left|X\left(f\right)\right|}^{2}}{S_{cc}\left(f\right){\left|X\left(f\right)\right|}^{2}+S_{nn}\left(f\right)}df$$

where the waveform energy is concentrated in the BW band. The expression of SINR is determined by the transmitted waveform, target ESV, noise PSD, and clutter PSD. 

To improve radar estimation performance on the target, the MI between the radar echo and the random target impulse response is utilized as the estimation criterion. The MI representation between the echo and the random target impulse response is [10]

(3) $$\;\mathrm{MI}\left({\left|X\left(f\right)\right|}^{2}\right)=Ty\int_{BW}\ln\left[1+\frac{\sigma_{H}{}^{2}\left(f\right){\left|X\left(f\right)\right|}^{2}}{T_{y}\left(S_{cc}\left(f\right){\left|X\left(f\right)\right|}^{2}+S_{nn}\left(f\right)\right)}\right]df$$

where Ty denotes the duration of the echo. The expression of MI is determined by the transmitted waveform, target ESV, noise PSD and clutter PSD. 

## 3. Fuzzy Signal Model and Problem Formulation

Due to the limitations of signal processing technology and the interference of noise in the real environment, the target and clutter spectra obtained by radar are imprecise. In this section, the model in [37] is introduced to describe the fuzziness of the target and clutter spectra. In the model, the target spectrum is considered to belong to the uncertainty class ε, which is restricted by the known upper and lower bounds, that is

(4) $$\left|H\left(f\right)\right|\in \varepsilon =\left\{l_{k}\leq \left|H\left(f_{k}\right)\right|\leq u_{k},\;\;\;\mathrm{for}\;k=1,2,\ldots ,K\right\}$$

**Figure 2:**
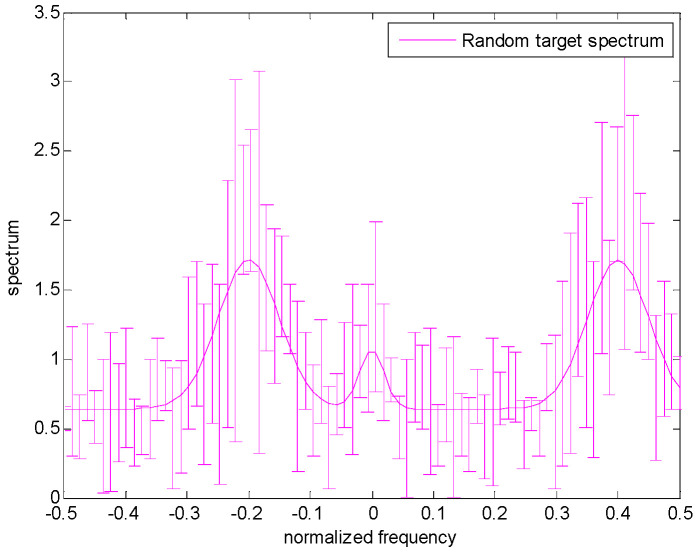
Model for the uncertain range of the random target spectrum.

Figure 2 shows the spectrum uncertainty range of the target. The real target spectrum is represented by a solid magenta line, and the upper and lower bounds of each frequency sample on the spectrum are represented by a margin of error, that is, the nominal value plus or minus a random number. Additionally, note that the differences between the upper and lower bounds could be varied at each frequency sample. Similarly, the clutter spectrum is also assumed to belong to the uncertainty class τ, which is expressed in Equation (5). Furthermore, the greater the difference between the upper bound and the lower bound, the more uncertain the clutter spectrum.

(5) $$\left|Scc\left(f\right)\right|\in \tau =\left\{d_{k}\leq \left|Scc\left(f_{k}\right)\right|\leq v_{k},\;\;\;\mathrm{for}\;k=1,2,\ldots ,K\right\}$$

### 3.1. Single-Robust Waveform Design Based on SINR and MI

In this subsection, the design method for the single-robust transmitted waveform based on the SINR and MI is considered. The two cases where the radar cannot accurately obtain the target and clutter spectra are considered separately. When the target spectrum and clutter spectrum are uncertain, there is an optimal transmitted scheme at each sampling frequency. So, the maximin robust transmitted waveform design techniques based on SINR and MI criteria are good approaches that ensure the performance of radar system in the worst case.

When the target spectrum is fuzzy, the maximin robust waveform design method should satisfy [33,37,38]

(6) $$\underset{{\left|X\left(f\right)\right|}^{2}}{\max}\left\{\underset{\left|H\left(f\right)\right|\epsilon \varepsilon }{\min}{\xi \left({\left|X\left(f\right)\right|}^{2},\sigma_{H}{}^{2}\left(f\right)\right)|}_{\int_{BW}{\left|X\left(f\right)\right|}^{2}df\leq Ex}\right\}$$

According to the theory of maximin single-robust signal processing [38], the solution to the maximin optimization problem is [33,38]

$${\xi \left({\left|X^{\mathrm{maxmin}}\left(f\right)\right|}^{2},\sigma_{H}{}^{2}\left(f\right)\right)|}_{\int_{BW}{\left|X^{\mathrm{maxmin}}\left(f\right)\right|}^{2}df\leq Ex}$$

(7) $$\geq {\xi \left({\left|X^{\mathrm{maxmin}}\left(f\right)\right|}^{2},\sigma_{H_{worst}}{}^{2}\left(f\right)\right)|}_{\int_{BW}{\left|X^{\mathrm{maxmin}}\left(f\right)\right|}^{2}df\leq Ex}$$

$$\geq {\xi \left({\left|X\left(f\right)\right|}^{2},\sigma_{H_{worst}}{}^{2}\left(f\right)\right)|}_{\int_{BW}{\left|X\left(f\right)\right|}^{2}df\leq Ex}$$

In the above Formula (7), for the right side of the inequality, the optimal transmitted waveform is the maximin optimal transmitted waveform when $\sigma_{H}{}^{2}\left(f\right)=\sigma_{H_{worst}}{}^{2}\left(f\right)$, which maximizes the performance of SINR of the matched filter and MI between radar echo and random target impulse response. If another transmitted waveform is utilized, the performance will be degraded. On the left side of the inequality implies that $\sigma_{H_{worst}}{}^{2}\left(f\right)$ is the worst target ESV corresponding to the maximin optimal transmitted waveform. In the uncertain range of the target spectrum, when maximin optimum transmitted waveform ${\left|X^{\mathrm{maxmin}}\left(f\right)\right|}^{2}$ is adopted, the performance of SINR and MI is better than that of the $\sigma_{H}{}^{2}\left(f\right)=\sigma_{H_{worst}}{}^{2}\left(f\right)$. In addition, the description of inequality (7) can be seen in the single-robust transmitted waveform design in reference [33]. It is also aimed at the situation of target spectrum uncertainty.

When the clutter spectrum is fuzzy, the maximin single-robust waveform design method should satisfy [37,38]

(8) $$\underset{{\left|X\left(f\right)\right|}^{2}}{\max}\left\{\underset{\left|Scc\left(f\right)\right|\epsilon \tau }{\min}{\xi \left({\left|X\left(f\right)\right|}^{2},Scc\left(f\right)\right)|}_{\int_{BW}{\left|X\left(f\right)\right|}^{2}df\leq Ex}\right\}$$

Based on the theory of maximin robust signal processing [38], the maximin optimization problem is solved as follows [33,38]:

(9) $$\begin{array}{c} {\xi \left({\left|X^{\mathrm{maxmin}}\left(f\right)\right|}^{2},Scc\left(f\right)\right)|}_{\int_{BW}{\left|X^{\mathrm{maxmin}}\left(f\right)\right|}^{2}df\leq Ex}\\ \geq {\xi \left({\left|X^{\mathrm{maxmin}}\left(f\right)\right|}^{2},Scc_{worst}\left(f\right)\right)|}_{\int_{BW}{\left|X^{\mathrm{maxmin}}\left(f\right)\right|}^{2}df\leq Ex}\\ \geq {\xi \left({\left|X\left(f\right)\right|}^{2},Scc_{worst}\left(f\right)\right)|}_{\int_{BW}{\left|X\left(f\right)\right|}^{2}df\leq Ex} \end{array}$$

In the above Formula (9), for the right side of the inequality, the optimal transmitted waveform is the maximin optimal transmitted waveform when $Scc\left(f\right)=Scc_{worst}\left(f\right)$. The performance will degrade if any other transmitted waveforms are employed. The $Scc_{worst}\left(f\right)$ on the left side of the inequality is the most unfavorable clutter PSD corresponding to the maximin optimal transmitted waveform. When the maximin optimal transmitted waveform ${\left|X^{\mathrm{maxmin}}\left(f\right)\right|}^{2}$ is used, the performance of SINR and MI is better than that of $Scc\left(f\right)=Scc_{worst}\left(f\right)$. For the proof of the inequalities above, see Appendix A.

#### 3.1.1. Single-Robust Waveform Design for the Fuzzy Target Spectrum

When the radar transmitted waveform is designed under the condition of a fuzzy target spectrum, the lower bound of the uncertain range of the target spectrum is taken as the target spectrum in the optimization problem, and where $\left|L\left(f\right)\right|=\left\{l_{k},k=1,2,\cdots ,K\right\}$ is the lower bound of the uncertain range of the target spectrum.

The maximin single-robust transmitted waveform optimization problem based on SINR under the condition of a fuzzy target spectrum should satisfy

(10) $$\underset{{\left|\tilde{X}\left(f\right)\right|}^{2}}{\max}\left\{\underset{\left|H\left(f\right)\right|\epsilon \varepsilon }{\min}{\mathrm{SIN}R\left({\left|\tilde{X}\left(f\right)\right|}^{2},\sigma_{H}{}^{2}\left(f\right)\right)|}_{\int_{BW}{\left|\tilde{X}\left(f\right)\right|}^{2}df\leq Ex}\right\}$$

The maximin single-robust waveform of the optimization problem (10) is

(11) $${\left|{\tilde{X}}^{\mathrm{maxmin}}\left(f\right)\right|}^{2}=\max\left[0,\;\;\tilde{B}\left(f\right)\left[\tilde{A}-\tilde{D}\left(f\right)\right]\right]$$

where

(12) $$\tilde{B}\left(f\right)=\frac{\sqrt{\sigma_{L}{}^{2}\left(f\right)S_{nn}\left(f\right)}}{Scc\left(f\right)}$$

(13) $$\tilde{D}\left(f\right)=\sqrt{\frac{S_{nn}\left(f\right)}{\sigma_{L}{}^{2}\left(f\right)}}$$

As shown in Equation (11), $\sigma_{L}{}^{2}\left(f\right)={\left|L\left(f\right)\right|}^{2}$ and $\tilde{A}$ is a constant determined by the constraint of energy:

(14) $$\int_{BW}\max\left[0,\tilde{B}\left(f\right)\left[\tilde{A}-\tilde{D}\left(f\right)\right]\right]df\leq Ex$$

The maximin single-robust transmitted waveform optimization problem based on MI under the condition of a fuzzy target spectrum should satisfy

(15) $$\underset{{\left|\bar{X}\left(f\right)\right|}^{2}}{\max}\left\{\underset{\left|H\left(f\right)\right|\epsilon \varepsilon }{\min}{MI\left({\left|\bar{X}\left(f\right)\right|}^{2},\sigma_{H}{}^{2}\left(f\right)\right)|}_{\int_{BW}{\left|\bar{X}\left(f\right)\right|}^{2}df\leq Ex}\right\}$$

The maximin single-robust waveform of the optimization problem (15) is

(16) $${\left|{\bar{X}}^{\mathrm{maxmin}}\left(f\right)\right|}^{2}=\max\left[0,\bar{B}\left(f\right)\left[\bar{A}-\bar{D}\left(f\right)\right]\right]$$

where

(17) $$\bar{B}\left(f\right)=\frac{\sigma_{L}{}^{2}\left(f\right)}{2TyS_{cc}\left(f\right)+\sigma_{L}{}^{2}\left(f\right)}$$

(18) $$\bar{D}\left(f\right)=\frac{TyS_{nn}\left(f\right)}{\sigma_{L}{}^{2}\left(f\right)}$$

and $\bar{A}$ denotes a constant determined by the constraint of energy:

(19) $$\int_{BW}\max\left[0,\bar{B}\left(f\right)\left[\bar{A}-\bar{D}\left(f\right)\right]\right]df\leq Ex$$

The above design method of a single-robust transmitted waveform based on SINR and MI is the same as that for single target spectrum uncertainty in reference [33]. This is also the basis of our research. Next, the case of clutter spectrum uncertainty is considered.

#### 3.1.2. Single-Robust Waveform Design for the Fuzzy Clutter Spectrum

When the radar transmitted waveform is designed under the condition of a fuzzy clutter spectrum, the upper bound of the uncertain range of the clutter spectrum is taken as the clutter spectrum in the optimization problem, and where $\left|V\left(f\right)\right|=\left\{v_{k},k=1,2,\cdots ,K\right\}$ is the upper bound of the uncertain range of the clutter spectrum.

The maximin single-robust transmitted waveform optimization problem based on SINR under the condition of a fuzzy clutter spectrum should satisfy.

(20) $$\underset{{\left|\overset{\approx }{X}\left(f\right)\right|}^{2}}{\max}\left\{\underset{\left|Scc\left(f\right)\right|\epsilon \tau }{\min}{\mathrm{SIN}R\left({\left|\overset{\approx }{X}\left(f\right)\right|}^{2},Scc\left(f\right)\right)|}_{\int_{BW}{\left|\overset{\approx }{X}\left(f\right)\right|}^{2}df\leq Ex}\right\}$$

The maximin single-robust waveform of the optimization problem (20) is

(21) $${\left|{\overset{\approx }{X}}^{\mathrm{maxmin}}\left(f\right)\right|}^{2}=\max\left[0,\overset{\approx }{B}\left(f\right)\left[\overset{\approx }{A}-\overset{\approx }{D}\left(f\right)\right]\right]$$

where

(22) $$\overset{\approx }{B}\left(f\right)=\frac{\sqrt{\sigma_{H}{}^{2}\left(f\right)S_{nn}\left(f\right)}}{V\left(f\right)}$$

(23) $$\overset{\approx }{D}\left(f\right)=\sqrt{\frac{S_{nn}\left(f\right)}{\sigma_{H}{}^{2}\left(f\right)}}$$

In Equation (21), $\overset{\approx }{A}$ is a constant defined by the constraint of energy:

(24) $$\int_{BW}\max\left[0\;,\overset{\approx }{B}\left(f\right)\left[\overset{\approx }{A}-\overset{\approx }{D}\left(f\right)\right]\right]df\leq Ex$$

The maximin single-robust transmitted waveform optimization problem based on MI under the condition of a fuzzy clutter spectrum should satisfy

(25) $$\underset{{\left|\overset{=}{X}\left(f\right)\right|}^{2}}{\max}\left\{\underset{\left|H\left(f\right)\right|\epsilon \varepsilon }{\min}{MI\left({\left|\overset{=}{X}\left(f\right)\right|}^{2},Scc\left(f\right)\right)|}_{\int_{BW}{\left|\overset{=}{X}\left(f\right)\right|}^{2}df\leq Ex}\right\}$$

The maximin single-robust waveform of the optimization problem (25) is

(26) $${\left|{\overset{=}{X}}^{\mathrm{maxmin}}\left(f\right)\right|}^{2}=\max\left[0,\overset{=}{B}\left(f\right)\left[\overset{=}{A}-\overset{=}{D}\left(f\right)\right]\right]$$

where

(27) $$\overset{=}{B}\left(f\right)=\frac{\sigma_{H}{}^{2}\left(f\right)}{2TyV\left(f\right)+\sigma_{H}{}^{2}\left(f\right)}$$

(28) $$\overset{=}{D}\left(f\right)=\frac{TyS_{nn}\left(f\right)}{\sigma_{H}{}^{2}\left(f\right)}$$

and $\overset{=}{A}$ is a constant defined by the constraint of energy:

(29) $$\int_{BW}\max\left[0,\overset{=}{B}\left(f\right)\left[\overset{=}{A}-\overset{=}{D}\left(f\right)\right]\right]df\leq Ex$$

### 3.2. Double-Robust Waveform Design Based on SINR and MI

In this subsection, considering that radar cannot simultaneously obtain precise target and clutter spectra, the design method for the double-robust transmitted waveform based on the SINR and MI is proposed. The SINR and MI optimization criteria for waveform design can be represented by $\xi \left({\left|X\left(f\right)\right|}^{2},\sigma_{H}{}^{2}\left(f\right),Scc\left(f\right)\right)$. So, the maximin double-robust waveform design method should satisfy

(30) $$\underset{{\left|X\left(f\right)\right|}^{2}}{\max}\left\{\underset{\left|H\left(f\right)\right|\epsilon \varepsilon ,\left|Scc\left(f\right)\right|\epsilon \tau }{\min}{\xi \left({\left|X\left(f\right)\right|}^{2},\sigma_{H}{}^{2}\left(f\right),Scc\left(f\right)\right)|}_{\int_{BW}{\left|X\left(f\right)\right|}^{2}df\leq Ex}\right\}$$

The double-robust transmitted waveform is designed under the condition that radar cannot simultaneously obtain precise target and clutter spectra. The lower bound of the uncertain range of the target spectrum is taken as the target spectrum, and the upper bound of the uncertain range of the clutter spectrum is denoted as the clutter spectrum in the optimization problem. The detailed design process is shown as follows.

#### 3.2.1. Double-Robust Waveform Design Based on SINR

The maximin double-robust transmitted waveform optimization problem based on SINR should satisfy

(31) $$\underset{{\left|\overset{≋}{X}\left(f\right)\right|}^{2}}{\max}\left\{\underset{\left|H\left(f\right)\right|\epsilon \varepsilon ,\left|Scc\left(f\right)\right|\epsilon \tau }{\min}{SINR\left({\left|\overset{≋}{X}\left(f\right)\right|}^{2},\sigma_{H}{}^{2}\left(f\right),Scc\left(f\right)\right)|}_{\int_{BW}{\left|\overset{≋}{X}\left(f\right)\right|}^{2}df\leq Ex}\right\}$$

The maximin double-robust waveform of the optimization problem (31) is

(32) $${\left|{\overset{≋}{X}}^{\mathrm{maxmin}}\left(f\right)\right|}^{2}=\max\left[0,\overset{≋}{B}\left(f\right)\left[\overset{≋}{A}-\overset{≋}{D}\left(f\right)\right]\right]$$

where

(33) $$\overset{≋}{B}\left(f\right)=\frac{\sqrt{\sigma_{L}{}^{2}\left(f\right)S_{nn}\left(f\right)}}{V\left(f\right)}$$

(34) $$\overset{≋}{D}\left(f\right)=\sqrt{\frac{S_{nn}\left(f\right)}{\sigma_{L}{}^{2}\left(f\right)}}$$

and $\overset{≋}{A}$ represents a constant which can be derived by the constraint of energy:

(35) $$\int_{BW}\max\left[0,\overset{≋}{B}\left(f\right)\left[\overset{≋}{A}-\overset{≋}{D}\left(f\right)\right]\right]df\leq Ex$$

In the above result of the double-robust transmitted waveform design based on SINR, by analyzing Equations (32)–(34), if we change the lower bound value of the target spectrum in the result to the value that can be accurately obtained, or if we change the upper bound value of the clutter spectrum in the result to the value that can be accurately obtained, then the double-robust transmitted waveform will degenerate into a single-robust transmitted waveform. The double-robust transmitted waveform design based on MI also has such characteristics.

In order to prove the conclusion above, the optimal problem should satisfy

(36) $$\begin{array}{c} {\mathrm{SIN}R\left({\left|{\overset{≋}{X}}^{\mathrm{maxmin}}\left(f\right)\right|}^{2},\sigma_{H}{}^{2}\left(f\right),Scc\left(f\right)\right)|}_{\int_{BW}{\left|{\overset{≋}{X}}^{\mathrm{maxmin}}\left(f\right)\right|}^{2}df\leq Ex}\\ \geq {\mathrm{SIN}R\left({\left|{\overset{≋}{X}}^{\mathrm{maxmin}}\left(f\right)\right|}^{2},\sigma_{H_{worst}}{}^{2}\left(f\right),Scc_{worst}\left(f\right)\right)|}_{\int_{BW}{\left|{\overset{≋}{X}}^{\mathrm{maxmin}}\left(f\right)\right|}^{2}df\leq Ex}\\ \geq {\mathrm{SIN}R\left({\left|\overset{≋}{X}\left(f\right)\right|}^{2},\sigma_{H_{worst}}{}^{2}\left(f\right),Scc_{worst}\left(f\right)\right)|}_{\int_{BW}{\left|\overset{≋}{X}\left(f\right)\right|}^{2}df\leq Ex} \end{array}$$

Assume that the lower bound of the target spectrum and the upper bound of the clutter spectrum can be obtained. The uncertainty of the target and clutter spectra are considered under certain energy constraints. To maximize the output SINR of the matched filter in the worst-case scenario, the double-robust transmitted waveform based on SINR is designed. We use the Lagrange multiplier method to solve the optimization model of the double-robust transmitted waveform. The main idea is to introduce a new parameter (Lagrange multiplier λ) between the objective function and the constraint condition to solve the optimization model.

The Lagrange multiplier method is employed to construct the objective function based on SINR:

(37) $$L\left({\left|\overset{≋}{X}\left(f\right)\right|}^{2},\lambda \right)=\int_{BW}\frac{\sigma_{L}{}^{2}\left(f\right){\left|\overset{≋}{X}\left(f\right)\right|}^{2}}{V\left(f\right){\left|\overset{≋}{X}\left(f\right)\right|}^{2}+S_{nn}\left(f\right)}df+\lambda \left(Ex-\int_{BW}{\left|\overset{≋}{X}\left(f\right)\right|}^{2}df\right)$$

When the target and clutter spectra are uncertain simultaneously, the output SINR of the matched filter is a function of ${\left|\overset{≋}{X}\left(f\right)\right|}^{2}$, the objective function (37) is also a function of ${\left|\overset{≋}{X}\left(f\right)\right|}^{2}$. Therefore, we can conceive of ${\left|\overset{≋}{X}\left(f\right)\right|}^{2}$ as an independent variable of the function (37), and $L\left({\left|\overset{≋}{X}\left(f\right)\right|}^{2}\right)$ as a dependent variable of the function (37). The expression (37) can be expressed as

(38) $$L\left({\left|\overset{≋}{X}\left(f\right)\right|}^{2},\lambda \right)=\int_{BW}\frac{\sigma_{L}{}^{2}\left(f\right){\left|\overset{≋}{X}\left(f\right)\right|}^{2}}{V\left(f\right){\left|\overset{≋}{X}\left(f\right)\right|}^{2}+S_{nn}\left(f\right)}df-\lambda \int_{BW}{\left|\overset{≋}{X}\left(f\right)\right|}^{2}df$$

This is equivalent to maximizing $L\left({\left|\overset{≋}{X}\left(f\right)\right|}^{2}\right)$ by solving ${\left|\overset{≋}{X}\left(f\right)\right|}^{2}$. In Equation (38), $L\left({\left|\overset{≋}{X}\left(f\right)\right|}^{2}\right)$ can be denoted by

(39) $$L\left({\left|\overset{≋}{X}\left(f\right)\right|}^{2}\right)=\frac{\sigma_{L}{}^{2}\left(f\right){\left|\overset{≋}{X}\left(f\right)\right|}^{2}}{V\left(f\right){\left|\overset{≋}{X}\left(f\right)\right|}^{2}+S_{nn}\left(f\right)}-\lambda {\left|\overset{≋}{X}\left(f\right)\right|}^{2}$$

Next, the first derivative of $L\left({\left|\overset{≋}{X}\left(f\right)\right|}^{2}\right)$ with respect to ${\left|\overset{≋}{X}\left(f\right)\right|}^{2}$ is given by

(40) $$\frac{dL\left({\left|\overset{≋}{X}\left(f\right)\right|}^{2}\right)}{d{\left|\overset{≋}{X}\left(f\right)\right|}^{2}}=\frac{\sigma_{L}{}^{2}\left(f\right)S_{nn}\left(f\right)}{{\left(V\left(f\right){\left|\overset{≋}{X}\left(f\right)\right|}^{2}+S_{nn}\left(f\right)\right)}^{2}}df-\lambda$$

Setting $\frac{dL\left({\left|\overset{≋}{X}\left(f\right)\right|}^{2}\right)}{d{\left|\overset{≋}{X}\left(f\right)\right|}^{2}}$ to zero yields the ${\left|\overset{≋}{X}\left(f\right)\right|}^{2}$ value which maximizes the output SINR, where ${\left|\overset{≋}{X}\left(f\right)\right|}^{2}$ is denoted by

(41) $${\left|\overset{≋}{X}\left(f\right)\right|}^{2}=-\frac{S_{nn}\left(f\right)}{V\left(f\right)}\pm \sqrt{\frac{\sigma_{L}{}^{2}\left(f\right)S_{nn}\left(f\right)}{\lambda {\left|V\left(f\right)\right|}^{2}}}$$

Let $\overset{≋}{A}=\sqrt{\frac{1}{\lambda }}$ ensure that ${\left|\overset{≋}{X}\left(f\right)\right|}^{2}$ is positive. Therefore, ${\left|\overset{≋}{X}\left(f\right)\right|}^{2}$ can be represented by

(42) $${\left|{\overset{≋}{X}}^{\mathrm{maxmin}}\left(f\right)\right|}^{2}=\max\left[0,\frac{\sqrt{\sigma_{L}{}^{2}\left(f\right)S_{nn}\left(f\right)}}{V\left(f\right)}\left(\overset{≋}{A}-\sqrt{\frac{S_{nn}\left(f\right)}{\sigma_{L}{}^{2}\left(f\right)}}\right)\right]$$

Equation (42) can also be written as

(43) $${\left|{\overset{≋}{X}}^{\mathrm{maxmin}}\left(f\right)\right|}^{2}=\max\left[0,\;\overset{≋}{B}\left(f\right)\left[\overset{≋}{A}-\overset{≋}{D}\left(f\right)\right]\right]$$

where

(44) $$\overset{≋}{B}\left(f\right)=\frac{\sqrt{\sigma_{L}{}^{2}\left(f\right)S_{nn}\left(f\right)}}{V\left(f\right)}$$

(45) $$\overset{≋}{D}\left(f\right)=\sqrt{\frac{S_{nn}\left(f\right)}{\sigma_{L}{}^{2}\left(f\right)}}$$

Therefore, we obtain the following:

$${\mathrm{SIN}R\left({\left|{\overset{≋}{X}}^{\mathrm{maxmin}}\left(f\right)\right|}^{2},\sigma_{H_{worst}}{}^{2}\left(f\right),Scc_{worst}\left(f\right)\right)|}_{\int_{BW}{\left|{\overset{≋}{X}}^{\mathrm{maxmin}}\left(f\right)\right|}^{2}df\leq Ex}$$

(46) $$\geq {\mathrm{SIN}R\left({\left|\overset{≋}{X}\left(f\right)\right|}^{2},\sigma_{H_{worst}}{}^{2}\left(f\right),Scc_{worst}\left(f\right)\right)|}_{\int_{BW}{\left|\overset{≋}{X}\left(f\right)\right|}^{2}df\leq Ex}$$

Then, we approximate the integral computation by substituting the spectrum results into the output SINR expression:

(47) $$\begin{array}{c} \mathrm{SIN}R\left({\left|{\overset{≋}{X}}^{\mathrm{maxmin}}\left(f\right)\right|}^{2},\sigma_{H}{}^{2}\left(f\right),Scc\left(f\right)\right)\\ =\overset{K}{\underset{k=1}{\sum }}∆f\frac{\sigma_{H}{}^{2}\left(f_{k}\right)\cdot \max\left[0,\frac{\sqrt{\sigma_{L}{}^{2}\left(f_{k}\right)S_{nn}\left(f_{k}\right)}}{V\left(f_{k}\right)}\left(\overset{≋}{A}-\sqrt{\frac{S_{nn}\left(f_{k}\right)}{\sigma_{L}{}^{2}\left(f_{k}\right)}}\right)\right]}{Scc\left(f_{k}\right)\cdot \max\left[0,\frac{\sqrt{\sigma_{L}{}^{2}\left(f_{k}\right)S_{nn}\left(f_{k}\right)}}{V\left(f_{k}\right)}\left(\overset{≋}{A}-\sqrt{\frac{S_{nn}\left(f_{k}\right)}{\sigma_{L}{}^{2}\left(f_{k}\right)}}\right)\right]+S_{nn}\left(f_{k}\right)}\\ \geq \overset{K}{\underset{k=1}{\sum }}∆f\frac{\sigma_{L}{}^{2}\left(f_{k}\right)\cdot \max\left[0,\frac{\sqrt{\sigma_{L}{}^{2}\left(f_{k}\right)S_{nn}\left(f_{k}\right)}}{V\left(f_{k}\right)}\left(\overset{≋}{A}-\sqrt{\frac{S_{nn}\left(f_{k}\right)}{\sigma_{L}{}^{2}\left(f_{k}\right)}}\right)\right]}{V\left(f_{k}\right)\cdot \max\left[0,\frac{\sqrt{\sigma_{L}{}^{2}\left(f_{k}\right)S_{nn}\left(f_{k}\right)}}{V\left(f_{k}\right)}\left(\overset{≋}{A}-\sqrt{\frac{S_{nn}\left(f_{k}\right)}{\sigma_{L}{}^{2}\left(f_{k}\right)}}\right)\right]+S_{nn}\left(f_{k}\right)}\\ =\mathrm{SIN}R\left({\left|{\overset{≋}{X}}^{\mathrm{maxmin}}\left(f\right)\right|}^{2},\sigma_{H_{worst}}{}^{2}\left(f\right),Scc_{worst}\left(f\right)\right) \end{array}$$

where, ∆f is the sampling interval. Consequently, we prove that $\sigma_{H_{worst}}{}^{2}\left(f\right)=\sigma_{L}{}^{2}\left(f\right)$ is the most unfavorable target ESV and $Scc_{worst}\left(f\right)=V\left(f\right)$ is the worst clutter PSD.

#### 3.2.2. Double-Robust Waveform Design Based on MI

The maximin double-robust transmitted waveform optimization problem based on MI should satisfy

(48) $$\underset{{\left|\overset{\equiv }{X}\left(f\right)\right|}^{2}}{\max}\left\{\underset{\left|H\left(f\right)\right|\epsilon \varepsilon ,\left|Scc\left(f\right)\right|\epsilon \tau }{\min}{MI\left({\left|\overset{\equiv }{X}\left(f\right)\right|}^{2},\sigma_{H}{}^{2}\left(f\right),Scc\left(f\right)\right)|}_{\int_{BW}{\left|\overset{\equiv }{X}\left(f\right)\right|}^{2}df\leq Ex}\right\}$$

The maximin double-robust waveform of the optimization problem (48) is

(49) $${\left|{\overset{\equiv }{X}}^{\mathrm{maxmin}}\left(f\right)\right|}^{2}=\max\left[0,\overset{\equiv }{B}\left(f\right)\left[\overset{\equiv }{A}-\overset{\equiv }{D}\left(f\right)\right]\right]$$

where

(50) $$\overset{\equiv }{B}=\frac{\sigma_{L}{}^{2}\left(f\right)}{2TyV\left(f\right)+\sigma_{L}{}^{2}\left(f\right)}$$

(51) $$\overset{\equiv }{D}=\frac{TyS_{nn}\left(f\right)}{\sigma_{H}{}^{2}\left(f\right)}$$

and $\overset{\equiv }{A}$ represents a constant which can be calculated by the constraint of energy:

(52) $$\int_{BW}\max\left[0,\overset{\equiv }{B}\left(f\right)\left[\overset{\equiv }{A}-\overset{\equiv }{D}\left(f\right)\right]\right]df\leq Ex$$

In order to prove the conclusion above, the optimal problem should satisfy

(53) $$\begin{array}{c} {MI\left({\left|{\overset{\equiv }{X}}^{\mathrm{maxmin}}\left(f\right)\right|}^{2},\sigma_{H}{}^{2}\left(f\right),Scc\left(f\right)\right)|}_{\int_{BW}{\left|{\overset{\equiv }{X}}^{\mathrm{maxmin}}\left(f\right)\right|}^{2}df\leq Ex}\\ \geq {MI\left({\left|{\overset{\equiv }{X}}^{\mathrm{maxmin}}\left(f\right)\right|}^{2},\sigma_{H_{worst}}{}^{2}\left(f\right),Scc_{worst}\left(f\right)\right)|}_{\int_{BW}{\left|{\overset{\equiv }{X}}^{\mathrm{maxmin}}\left(f\right)\right|}^{2}df\leq Ex}\\ \geq {MI\left({\left|\overset{\equiv }{X}\left(f\right)\right|}^{2},\sigma_{H_{worst}}{}^{2}\left(f\right),Scc_{worst}\left(f\right)\right)|}_{\int_{BW}{\left|\overset{\equiv }{X}\left(f\right)\right|}^{2}df\leq Ex} \end{array}$$

The double-robust transmitted waveform based on MI is designed to maximize the MI between radar echo and random target impulse response in the worst-case scenario. The Lagrange multiplier method is also used to solve the optimization model.

The Lagrange multiplier method is employed to construct the objective function based on MI:

(54) $$L\left({\left|\overset{\equiv }{X}\left(f\right)\right|}^{2},\lambda \right)=Ty\int_{BW}\ln\left[1+\frac{\sigma_{L}{}^{2}\left(f\right){\left|\overset{\equiv }{X}\left(f\right)\right|}^{2}}{T_{y}\left(V\left(f\right){\left|\overset{\equiv }{X}\left(f\right)\right|}^{2}+S_{nn}\left(f\right)\right)}\right]df+\lambda \left(Ex-\int_{BW}{\left|\overset{\equiv }{X}\left(f\right)\right|}^{2}df\right)$$

When the target and clutter spectra are uncertain simultaneously, the MI between radar echo and random target impulse response is a function of ${\left|\overset{\equiv }{X}\left(f\right)\right|}^{2}$, the objective function (54) is also a function of ${\left|\overset{\equiv }{X}\left(f\right)\right|}^{2}$. Therefore, we can conceive of ${\left|\overset{\equiv }{X}\left(f\right)\right|}^{2}$ as an independent variable of the function and $L\left({\left|\overset{\equiv }{X}\left(f\right)\right|}^{2}\right)$ as a dependent variable of the function. Equation (54) can be expressed as

(55) $$L\left({\left|\overset{\equiv }{X}\left(f\right)\right|}^{2},\lambda \right)=Ty\int_{BW}\ln\left[1+\frac{\sigma_{L}{}^{2}\left(f\right){\left|\overset{\equiv }{X}\left(f\right)\right|}^{2}}{T_{y}\left(V\left(f\right){\left|\overset{\equiv }{X}\left(f\right)\right|}^{2}+S_{nn}\left(f\right)\right)}\right]df-\lambda \int_{BW}{\left|\overset{\equiv }{X}\left(f\right)\right|}^{2}df$$

This is equivalent to maximizing $L\left({\left|\overset{\equiv }{X}\left(f\right)\right|}^{2}\right)$ by solving ${\left|\overset{\equiv }{X}\left(f\right)\right|}^{2}$. In Equation (55), $L\left({\left|\overset{\equiv }{X}\left(f\right)\right|}^{2}\right)$ can be denoted by

(56) $$L\left({\left|\overset{\equiv }{X}\left(f\right)\right|}^{2}\right)=Ty\ln\left[1+\frac{\sigma_{L}{}^{2}\left(f\right){\left|\overset{\equiv }{X}\left(f\right)\right|}^{2}}{T_{y}\left(V\left(f\right){\left|\overset{\equiv }{X}\left(f\right)\right|}^{2}+S_{nn}\left(f\right)\right)}\right]df-\lambda {\left|\overset{\equiv }{X}\left(f\right)\right|}^{2}df$$

Next, the first derivative of $L\left({\left|\overset{≋}{X}\left(f\right)\right|}^{2}\right)$ with respect to ${\left|\overset{≋}{X}\left(f\right)\right|}^{2}$ is obtained, and making the derivative equal to zero yields

(57) $$\lambda =\frac{\sigma_{L}{}^{2}\left(f\right)S_{nn}\left(f\right)}{A\left(f\right){\left|\overset{\equiv }{X}\left(f\right)\right|}^{4}+E\left(f\right){\left|\overset{\equiv }{X}\left(f\right)\right|}^{2}+C\left(f\right)}$$

where A(f),E(f), and C(f) are simplified alternatives:

(58) $$A\left(f\right)=\frac{V\left(f\right)\left(TyV\left(f\right)+\sigma_{L}{}^{2}\left(f\right)\right)}{Ty}$$

(59) $$E\left(f\right)=\frac{S_{nn}\left(f\right)\left(2TyV\left(f\right)+\sigma_{L}{}^{2}\left(f\right)\right)}{Ty}$$

(60) $$C\left(f\right)={\left|S_{nn}\left(f\right)\right|}^{2}$$

Let $\overset{\equiv }{A}=\frac{Ty}{\lambda }$, $\overset{\equiv }{A}$ is positive because λ is greater than zero. Therefore, ${\left|\overset{\equiv }{X}\left(f\right)\right|}^{2}$ is guaranteed to be positive and can be represented as

(61) $${\left|{\overset{\equiv }{X}}^{\mathrm{maxmin}}\left(f\right)\right|}^{2}=\max\left[0,\;-\overset{\equiv }{R}\left(f\right)+\sqrt{{\overset{\equiv }{R}}^{2}\left(f\right)+\overset{\equiv }{S}\left(f\right)\left(\overset{\equiv }{A}-\overset{\equiv }{D}\left(f\right)\right)}\right]$$

where

(62) $$\overset{\equiv }{R}\left(f\right)=\frac{S_{nn}\left(f\right)\left(2TyV\left(f\right)+\sigma_{L}{}^{2}\left(f\right)\right)}{2V\left(f\right)\left(TyV\left(f\right)+\sigma_{L}{}^{2}\left(f\right)\right)}$$

(63) $$\overset{\equiv }{D}\left(f\right)=\frac{S_{nn}\left(f\right)}{Ty\sigma_{L}{}^{2}\left(f\right)}$$

(64) $$\overset{\equiv }{S}\left(f\right)=\frac{S_{nn}\left(f\right)\sigma_{L}{}^{2}\left(f\right)}{V\left(f\right)\left(TyV\left(f\right)+\sigma_{L}{}^{2}\left(f\right)\right)}$$

The expression (61) is further expressed as

(65) $${\left|{\overset{\equiv }{X}}^{\mathrm{maxmin}}\left(f\right)\right|}^{2}=\max\left[0,\overset{\equiv }{B}\left(f\right)\left[\overset{\equiv }{A}-\overset{\equiv }{D}\left(f\right)\right]\right]$$

where

(66) $$\overset{\equiv }{B}=\frac{\sigma_{L}{}^{2}\left(f\right)}{2TyV\left(f\right)+\sigma_{L}{}^{2}\left(f\right)}$$

(67) $$\overset{\equiv }{D}=\frac{TyS_{nn}\left(f\right)}{\sigma_{H}{}^{2}\left(f\right)}$$

Therefore, we obtain the following:

$${MI\left({\left|{\overset{\equiv }{X}}^{\mathrm{maxmin}}\left(f\right)\right|}^{2},\sigma_{H_{worst}}{}^{2}\left(f\right),Scc_{worst}\left(f\right)\right)|}_{\underset{BW}{\int }{\left|{\overset{\equiv }{X}}^{\mathrm{maxmin}}\left(f\right)\right|}^{2}df\leq Ex}$$

(68) $$\geq {MI\left({\left|\overset{\equiv }{X}\left(f\right)\right|}^{2},\sigma_{H_{worst}}{}^{2}\left(f\right),Scc_{worst}\left(f\right)\right)|}_{\int_{BW}{\left|\overset{\equiv }{X}\left(f\right)\right|}^{2}df\leq Ex}$$

Then, we approximate the integral computation by substituting the spectrum results into the MI expression:

$$MI\left({\left|{\overset{\equiv }{X}}^{\mathrm{maxmin}}\left(f\right)\right|}^{2},\sigma_{H}{}^{2}\left(f\right),Scc\left(f\right)\right)$$

$$=Ty\overset{K}{\underset{k=1}{\sum }}∆f\cdot \ln\left[1+\frac{\sigma_{H}{}^{2}\left(f_{k}\right){\left|{\overset{\equiv }{X}}^{\mathrm{maxmin}}\left(f_{k}\right)\right|}^{2}}{T_{y}\left(S_{cc}\left(f_{k}\right){\left|{\overset{\equiv }{X}}^{\mathrm{maxmin}}\left(f_{k}\right)\right|}^{2}+S_{nn}\left(f_{k}\right)\right)}\right]$$

(69) $$=Ty\overset{K}{\underset{k=1}{\sum }}∆f\cdot \ln\left[1+\frac{\sigma_{H}{}^{2}\left(f_{k}\right)\max\left[0,\overset{\equiv }{B}\left(f_{k}\right)\left[\overset{\equiv }{A}-\overset{\equiv }{D}\left(f_{k}\right)\right]\right]}{T_{y}\left(S_{cc}\left(f_{k}\right)\max\left[0,\overset{\equiv }{B}\left(f_{k}\right)\left[\overset{\equiv }{A}-\overset{\equiv }{D}\left(f_{k}\right)\right]\right]+S_{nn}\left(f_{k}\right)\right)}\right]$$

$$\geq Ty\overset{K}{\underset{k=1}{\sum }}∆f\cdot \ln\left[1+\frac{\sigma_{L}{}^{2}\left(f_{k}\right)\max\left[0,\overset{\equiv }{B}\left(f_{k}\right)\left[\overset{\equiv }{A}-\overset{\equiv }{D}\left(f_{k}\right)\right]\right]}{T_{y}\left(V\left(f_{k}\right)\max\left[0,\overset{\equiv }{B}\left(f_{k}\right)\left[\overset{\equiv }{A}-\overset{\equiv }{D}\left(f_{k}\right)\right]\right]+S_{nn}\left(f_{k}\right)\right)}\right]$$

$$=MI\left({\left|{\overset{\equiv }{X}}^{\mathrm{maxmin}}\left(f\right)\right|}^{2},\sigma_{H_{worst}}{}^{2}\left(f\right),Scc_{worst}\left(f\right)\right)$$

From Equation (69), $\sigma_{H_{worst}}{}^{2}\left(f\right)=\sigma_{L}{}^{2}\left(f\right)$ is the most unfavorable target ESV and $Scc_{worst}\left(f\right)=V\left(f\right)$ is the most disadvantageous clutter PSD. So that completes the proof.

## 4. Simulation and Results

The simulation results in this section demonstrate the effectiveness and practicability of the double-robust transmitted waveforms based on SINR and MI criteria.

**Figure 3:**
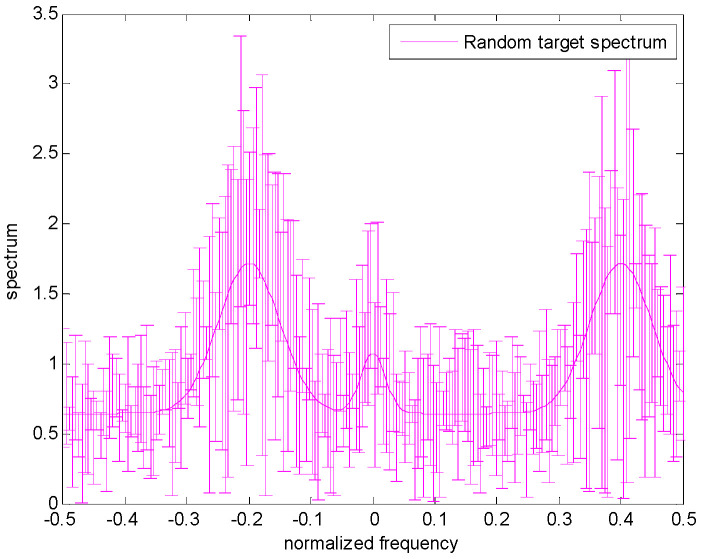
Uncertainty model for target spectrum.

**Figure 4:**
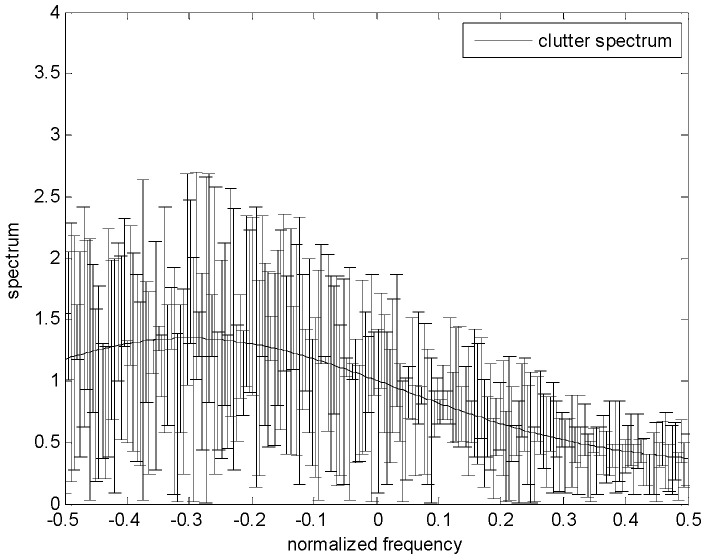
Uncertainty model for clutter spectrum.

The uncertain ranges of the target spectrum and clutter spectrum are shown in Figure 3 and Figure 4, respectively. The precise target spectrum and clutter spectrum, which cannot be obtained by the radar system in an actual scene, are represented by solid lines. The upper and lower bounds at each sampling are represented by the deviation bounds. The upper bound is the precise target spectrum or clutter spectrum plus a random value. Similarly, the lower bound is the precise target spectrum or clutter spectrum minus a random value. The main simulation parameters are shown in Table 1.

**Figure 5:**
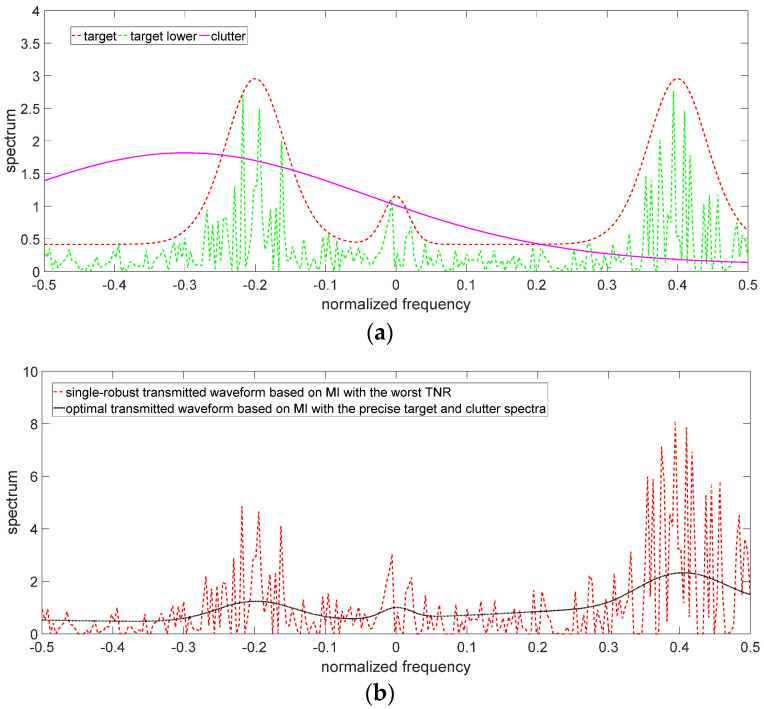

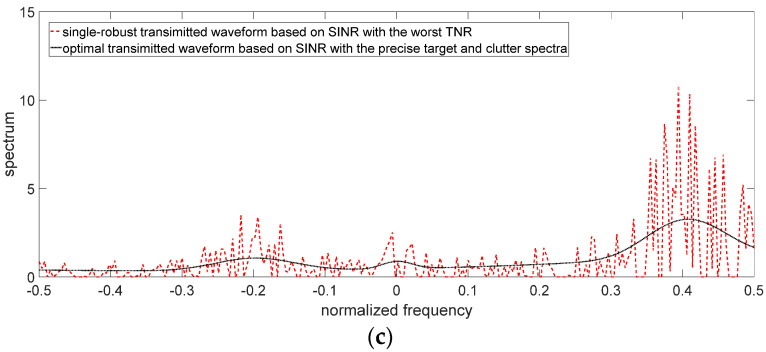
Single-robust waveforms with the uncertain target spectrum. (**a**) Target and clutter spectra in the case of the worst TNR. (**b**) Single-robust transmitted waveform based on MI. (**c**) Single-robust transmitted waveform based on SINR.

**Figure 6:**
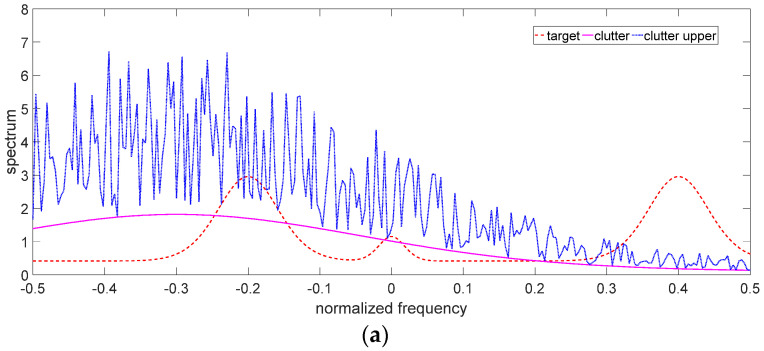

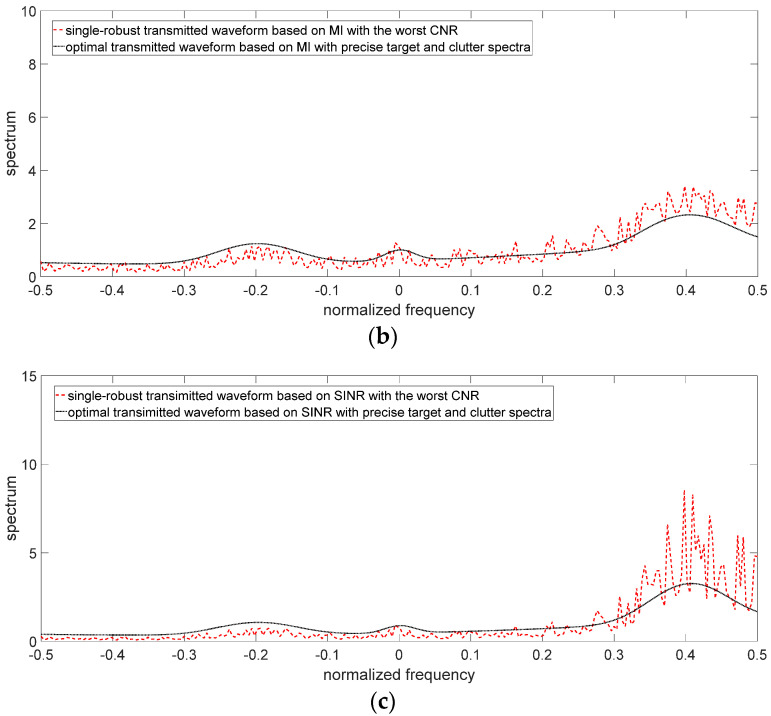
Single-robust waveforms with the uncertain clutter spectrum. (**a**) Target and clutter spectra in the case of the worst CNR. (**b**) Single-robust transmitted waveform based on MI. (**c**) Single-robust transmitted waveform based on SINR.

**Figure 7:**
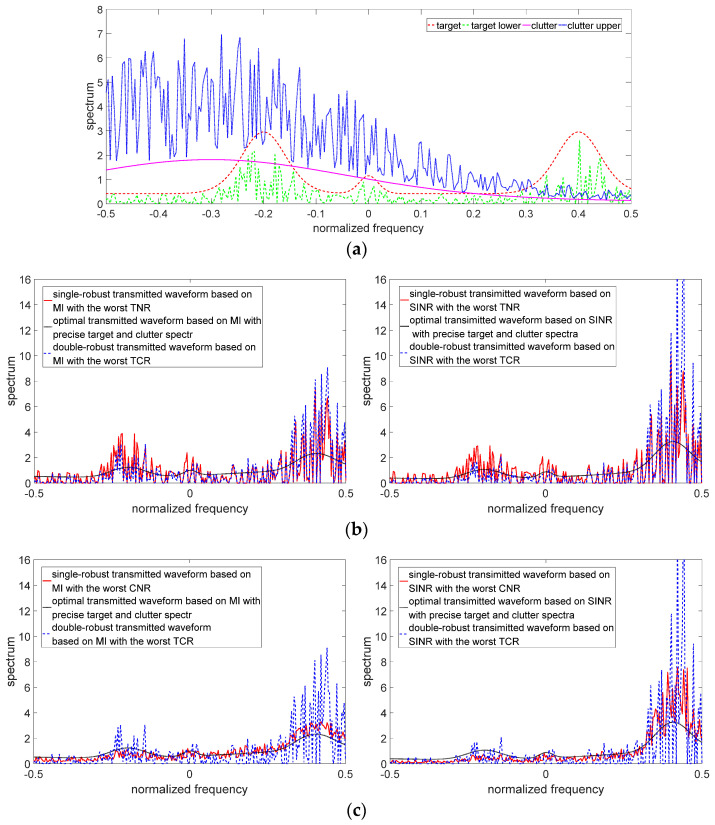
Double-robust waveforms with the uncertain target and clutter spectra. (**a**) Target and clutter spectra in the case of the worst TCR. (**b**) Double-robust transmitted waveform vs. single-robust waveforms with the worst TNR. (**c**) Double-robust transmitted waveform vs. single-robust waveforms with the worst CNR.

The results of the robust waveform designs (including single-robust waveforms and double-robust waveforms) based on SINR and MI criteria are depicted in Figure 5, Figure 6 and Figure 7. Since the robust waveform designs consider the worst cases, the lower bound of the uncertain target spectrum and/or the upper bound of the uncertain clutter spectrum are used to design the robust transmitted waveforms. The optimal transmitted waveforms (OTWs) based on SINR and MI criteria at the best case are adopted as a benchmark.

Figure 5 depicts the results of the single-robust transmitted waveforms (SRWs) based on MI and SINR criteria when the target spectrum is uncertain. The SRWs in the case of the worst TNR have the following characteristics:

(1) The amplitudes of the SRWs fluctuate around those of the OTWs. The two types of waveforms have the same energy allocation strategy. The SRWs with the worst CNR and the double-robust waveforms with the worst TCR also have the same characteristics (see Figure 6b,c and Figure 7b,c), and because they are designed based on the same criteria, they can allocate the majority of energy to the sub-frequency band with the strong target spectrum.

(2) In the case of the worst TNR, the SRWs need to allocate more energy than the OTWs in the sub-frequency band where the target spectrum is strong. Since the radar cannot obtain the precise target spectrum in the worst TNR, it can only use the lower bound of the uncertain target spectrum to optimize the transmitted waveforms, which leads to the SRWs needing to allocate more energy than OTWs in the sub-frequency band with the strong target spectrum to ensure the improvement in MI and SINR, such as the energy allocation in the sub-frequency bands around the central frequencies of −0.2, 0, and 0.4 in Figure 5b,c.

Figure 6 describes the results of the single-robust transmitted waveforms based on MI and SINR criteria when the clutter spectrum is uncertain. The SRWs in the case of the worst CNR have the following characteristics:

(1) The SRWs allocate the most energy to the sub-frequency bands where the upper bound of the uncertain clutter spectrum (the blue line in Figure 6a) is less than the target spectrum, which could enhance the improvement in MI and SINR in the worst CNR, such as the energy allocation in the sub-frequency bands around the central frequencies −0.2, 0, and 0.4 in Figure 6b,c.

(2) The energy allocation of SRWs is more than that of OTWs in the sub-frequency band where the upper bound of the uncertain clutter spectrum is less than the target spectrum. Since the precise clutter spectrum cannot be obtained by radar in an actual scene, radar can only use the data in the upper bound of the uncertain clutter spectrum in the worst case; thus, the result is worse than it actually is. Meanwhile, the target spectrum is greater than the upper bound of the uncertain clutter spectrum. SRWs must allocate more energy than OTWs to ensure the improvement in MI and SINR, such as the energy allocation in the sub-frequency band around the central frequency of 0.4 in Figure 6b,c.

(3) The energy allocation of the SRWs is less than that of the OTWs in the sub-frequency bands where the upper bound of the uncertain clutter spectrum is greater than the target spectrum. This energy allocation strategy of SRWs is still aimed at maximizing SINR and MI in the worst CNR, because when the upper bound of the uncertain clutter spectrum is much greater than the target spectrum in some sub-frequency bands, it means that the clutter echo interferes with the target echo more. Therefore, in these sub-frequency bands, the energy allocation of the SRWs is less than that of the OTWs to reduce the SINR and MI loss. Such as the energy allocation in the sub-frequency bands around the central frequency of −0.2 in Figure 6b,c.

Figure 7 describes the results of the double-robust transmitted waveforms (DRWs) based on MI and SINR criteria with simultaneous uncertainty of the target and clutter spectra. The DRWs in the case of the worst TCR have the following characteristics:

(1) The DRWs also allocate the majority of energy to the sub-frequency bands with strong target spectrum features.

(2) MI-based DRW allocates less energy than SINR-based DRW in the sub-frequency band where the upper bound of the uncertain clutter spectrum is less than the lower bound of the uncertain target spectrum. On the contrary, MI-based DRW allocates more energy than SINR-based DRW in the sub-frequency band where the upper bound of the uncertain clutter spectrum is greater than the lower bound of the uncertain target spectrum. This is because MI as an optimization criterion makes radar pay more attention to the acquisition of the target information than the influence of clutter on the radar.

(3) Compared with the SRWs with the worst TNR, the DRWs allocate more energy than SRWs in the sub-frequency band where the upper bound of the uncertain clutter spectrum is less than the lower bound of the uncertain target spectrum. On the contrary, the DRWs allocate less energy than SRWs in the sub-frequency band where the upper bound of the uncertain clutter spectrum is greater than the lower bound of the uncertain target spectrum. Such as the energy allocation in the sub-frequency bands around the central frequencies of −0.2, 0, and 0.4 in Figure 7b. This is because the DRWs not only consider the uncertain target spectrum, but also consider the influence of the clutter spectrum on MI and SINR, while the SRWs only consider the uncertain target spectrum.

(4) Compared with the SRWs with the worst CNR, the DRWs allocate more energy than the SRWs in the sub-frequency bands with strong target spectrum features regardless of whether the lower bound of the uncertain target spectrum is greater than the upper bound of the uncertain clutter spectrum, such as the energy allocation in the sub-frequency bands around the central frequencies of −0.2, 0, and 0.4 in Figure 7c. This is also because the DRWs consider not only the uncertain target spectrum but also the influence of the clutter spectrum on MI and SINR, while the SRWs only consider the uncertain clutter spectrum.

**Figure 8:**
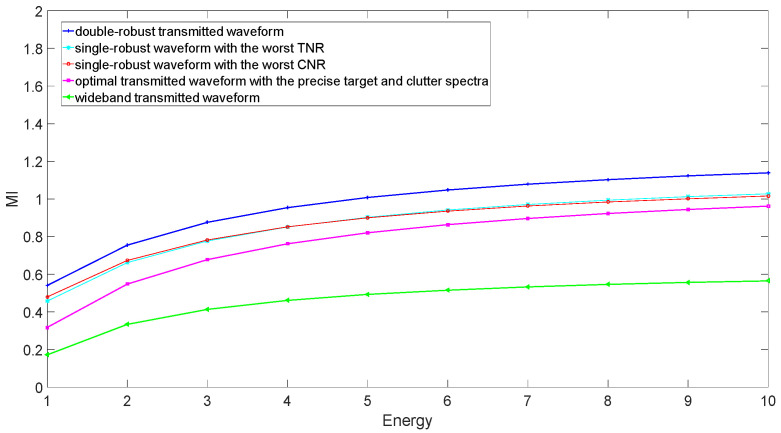
MI performance corresponding to different radar transmitted waveforms in the case of the worst TCR.

**Figure 9:**
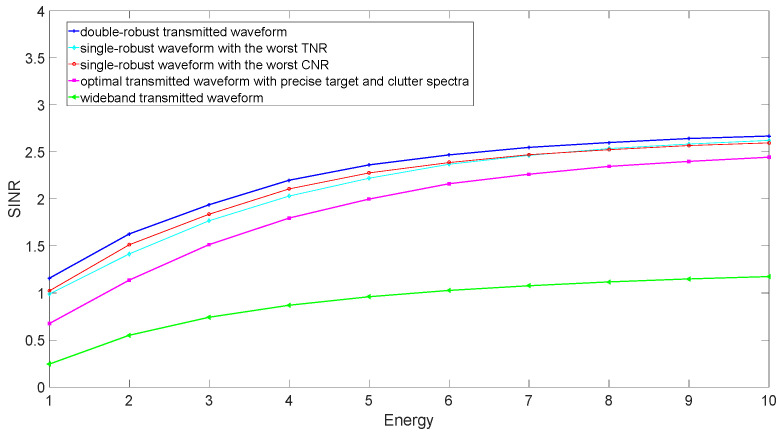
SINR performance corresponding to different radar transmitted waveforms in the case of the worst TCR.

Assume that the total energy of the transmitted waveform increases from 1 to 10 J. The MIs and SINRs corresponding to the several transmitted waveforms are compared in the case of the worst TCR in Figure 8 and Figure 9, respectively. The wideband waveform is used as a benchmark, which allocates the transmitted energy uniformly across the whole frequency band. The wideband waveform has the worst MI and SINR because of a lack of information about target and clutter. OTWs perform better than wideband waveform, because OTWs allocate energy according to precise information about the target and clutter, although this information is not captured in an actual scene. The SRWs have better MI and SINR than the OTWs, because they can use the lower bound of the uncertain target spectrum or the upper bound of the uncertain clutter spectrum in the case of the worst TCR. The best MI and SINR can be obtained when utilizing the DRWs in the case of the worst TCR. That is due to the fact that the uncertain target and clutter spectra in an actual scene are considered in the double-robust waveform design scheme.

## 5. Conclusions

In this paper, the double-robust transmitted waveform design based on SINR and MI is proposed in the presence of clutter, respectively. First, the single-robust transmitted waveforms are designed for the worst TNR and CNR cases. The single-robust transmitted waveforms with the worst TRN allocate more energy than the optimal transmitted waveforms with the best case in the sub-frequency band where the target spectrum is strong. The single-robust waveforms with the worst CNR allocate more energy than the optimal transmitted waveforms with the best case when the target spectrum is greater than the upper bound of the uncertain clutter spectrum. On the contrary, they allocate less energy than the optimal transmitted waveforms with the best case when the target spectrum is less than the upper bound of the uncertain clutter spectrum. All single-robust waveforms allocate the majority of energy to the sub-frequency bands with strong target spectrum features. Since radar cannot obtain the precise target and clutter spectrum in an actual scene, the double-robust waveforms are proposed in the case of the worst TCR. The double-robust waveforms also allocate the majority of energy to the sub-frequency bands with strong target spectrum features. However, they can further improve the MI and SINR compared to the single-robust waveforms. Finally, the simulation results show that the double-robust transmitted waveforms can maximize SINR and MI. In the case of the worst TCR, the performance of SINR and MI will degrade if other transmitted waveforms are employed.

## 6. Future Research

The communication base station (BS) will be considered to be added to the existing model to form a joint radar and communication system. The orthogonal frequency division multiplexing (OFDM) waveforms with N subcarriers will be considered to be used for radar and communication base stations to design the radar transmitted waveform.

## Figures and Tables

**Table 1:** Main simulation parameters.

Parameter	Value
Noise PSD	1 W/HZ
TNR ^1^ with the precise target and clutter spectra (the best case)	−0.31 dB
TNR with the uncertain target spectrum (the worst case)	−4.39 dB
CNR ^2^ with the precise target and clutter spectra (the best case)	−0.29 dB
CNR with the uncertain clutter spectrum (the worst case)	3.78 dB
TCR ^3^ with the precise target and clutter spectra (the best case)	−0.02 dB
TCR with the uncertain target and clutter spectra (the worst case)	−8.18 dB
Transmitted signal energy	1 J
Sampling points	256
Ty	1 s

^1^ The target-to-noise ratio (TNR) [39]. ^2^ The clutter-to-noise ratio (CNR) [39]. ^3^ The target-to-clutter ratio (TCR) [39].

## Data Availability

Data is available upon reasonable request from the authors.

## Appendix A

In [38], the minimax robust design formulas are provided.

(A1) $$\underset{\ell }{\min}\;\underset{f\epsilon F}{\max}\;V\left(\ell ,f\right)$$

(A2) $$V\left(\ell_{R},f\right)\leq V\left(\ell_{R},f_{R}\right)\leq V\left(\ell ,f_{R}\right)$$

(A1) and (A2) provided the maxmin robust design. The existence of $\left(\ell_{R},f_{R}\right)$ is equivalent to the following conditions:

(A3) $$\underset{\ell }{\min}\;\underset{f\epsilon F}{\max}\;V\left(\ell ,f\right)=\underset{f\epsilon F}{\max}\;\underset{\ell }{\min}\;V\left(\ell ,f\right)$$

In [37], The optimization criterion for robust MIMO radar waveform design is

(A4) $$\underset{T}{\min}\left\{\underset{f\epsilon F}{\max}{\xi \left(T,f\right)|}_{tr\left\{T\right\}\leq LQP_{0}}\right\}$$

where, $T=x^{H}x$, x is the MIMO radar waveform covariance matrix. $LQP_{0}$ is the total energy of the transmitted signal in [37].

According to the minimax robust signal processing theory in [38], reference [37] found $T=T_{L}$ that satisfies the saddle point conditions

$${\xi \left(T_{L},f\right)|}_{tr\left\{T_{L}\right\}\leq LQP_{0}}\leq {\xi \left(T_{L},f_{L}\right)|}_{tr\left\{T_{L}\right\}\leq LQP_{0}}$$

(A5) $$\;\;\;\;\;\;\;\;\;\;\;\;\;\;\;\;\;\;\;\;\;\;\;\;\;\;\;\;\;\;\;\;\;\;\;\;\;\;\;\;\;\;\;\;\;\;\;\;\;\;\;\;\;\;\;\;\;\;\;\;\;\;\;\;\leq {\xi \left(T,f_{L}\right)|}_{tr\left\{T\right\}\leq LQP_{0}}\;\;\;\;\;\;\;$$

where, $f_{L}$ denotes the least favorable PSD, and $T_{L}$ denotes the minimax robust waveform covariance matrix. According to [38], we can get

(A6) $$\underset{T}{\min}\left\{\underset{f\epsilon F}{\max}{\xi \left(T,f\right)|}_{tr\left\{T\right\}\leq LQP_{0}}\right\}=\underset{f\epsilon F}{\max}\left\{\underset{T}{\min}{\xi \left(T,f\right)|}_{tr\left\{T\right\}\leq LQP_{0}}\right\}$$

In this paper, we take the single-robust transmitted waveform design as an example under the uncertain clutter spectrum. SISO radar could be regarded as a special case of MIMO radar, when both transmit and receive antennas are 1. Then, we have $T={\left|X\left(f\right)\right|}^{2}$, fϵF=|Scc(f)|ϵτ, $T_{L}={\left|X^{maxmin}\left(f\right)\right|}^{2}$, and $f_{L}=Scc_{worst}\left(f\right)$.

We set

(A7) $$\underset{{\left|X\left(f\right)\right|}^{2}}{\max}\left\{\underset{\left|Scc\left(f\right)\right|\epsilon \tau }{\min}{\xi \left({\left|X\left(f\right)\right|}^{2},Scc\left(f\right)\right)|}_{\int_{BW}{\left|X\left(f\right)\right|}^{2}df\leq Ex}\right\}$$

Then, according toreference [38], we can get

$$\underset{{\left|X\left(f\right)\right|}^{2}}{\max}\left\{\underset{\left|Scc\left(f\right)\right|\epsilon \tau }{\min}{\xi \left({\left|X\left(f\right)\right|}^{2},Scc\left(f\right)\right)|}_{\int_{BW}{\left|X\left(f\right)\right|}^{2}df\leq Ex}\right\}$$

(A8) $$=\underset{\left|Scc\left(f\right)\right|\epsilon \tau }{\min}\left\{\underset{{\left|X\left(f\right)\right|}^{2}}{\max}{\xi \left({\left|X\left(f\right)\right|}^{2},Scc\left(f\right)\right)|}_{\int_{BW}{\left|X\left(f\right)\right|}^{2}df\leq Ex}\right\}$$

According to [37,38], from the above formulas, we can obtain

$${\xi \left({\left|X\left(f\right)\right|}^{2},Scc_{worst}\left(f\right)\right)|}_{\int_{BW}{\left|X\left(f\right)\right|}^{2}df\leq Ex}$$

(A9) $$\leq {\xi \left({\left|X^{\mathrm{maxmin}}\left(f\right)\right|}^{2},Scc_{worst}\left(f\right)\right)|}_{\int_{BW}{\left|X^{\mathrm{maxmin}}\left(f\right)\right|}^{2}df\leq Ex}$$

$$\leq {\xi \left({\left|X^{\mathrm{maxmin}}\left(f\right)\right|}^{2},Scc\left(f\right)\right)|}_{\int_{BW}{\left|X^{\mathrm{maxmin}}\left(f\right)\right|}^{2}df\leq Ex}$$

After sorting we get

$${\xi \left({\left|X^{\mathrm{maxmin}}\left(f\right)\right|}^{2},Scc\left(f\right)\right)|}_{\int_{BW}{\left|X^{\mathrm{maxmin}}\left(f\right)\right|}^{2}df\leq Ex}$$

(A10) $$\geq {\xi \left({\left|X^{\mathrm{maxmin}}\left(f\right)\right|}^{2},Scc_{worst}\left(f\right)\right)|}_{\int_{BW}{\left|X^{\mathrm{maxmin}}\left(f\right)\right|}^{2}df\leq Ex}$$

$$\geq {\xi \left({\left|X\left(f\right)\right|}^{2},Scc_{worst}\left(f\right)\right)|}_{\int_{BW}{\left|X\left(f\right)\right|}^{2}df\leq Ex}$$
